# The Effects of Motor Imagery on Trapeziometacarpal Osteoarthritis in Women During the Post-Surgical Immobilization Period: A Randomized Clinical Trial

**DOI:** 10.3390/healthcare13091011

**Published:** 2025-04-28

**Authors:** Eva Prado-Robles, Jose Ángel Delgado-Gil, Jesús Seco-Calvo

**Affiliations:** 1 Unit of Physical Medicine and Rehabilitation, León University Hospital, Castilla y León Health Service, 24008 León, Spain; 2 Biosanitary Research Institute (IBioLEÓN), 24071 León, Spain; jadelgado@saludcastillayleon.es; 3 Primary Care Service Area, Castilla y León Health Service, 24008 León, Spain; 4 Institute of Biomedicine (IBIOMED), Universidad de León, 24071 León, Spain

**Keywords:** mental practice, motor imagery, occupational therapy, trapeziometacarpal osteoarthritis, immobilization, hand surgery

## Abstract

Trapeziometacarpal osteoarthritis is the second most frequent degenerative hand disease, and it presents in 66% of women over the age of 55. Post-surgery immobilization results in functional losses that could be attenuated by motor imagery training. **Objectives**: The aim of this study is to evaluate the efficacy of motor imagery training during the post-surgical immobilization period in women who underwent surgery for trapeziometacarpal osteoarthritis. **Methods**: A randomized controlled trial was performed. A total of 40 patients satisfied the eligibility criteria, agreed to participate, and were randomized into an experimental group (n = 20) or control group (n = 20). Motor imagery was applied to the experimental group during the 3 weeks of post-surgical immobilization and to the control group with the conventional protocol. Measurement outcomes were assessed four times throughout the study using the Disabilities of the Arm, Shoulder, and Hand questionnaire, the Cochin Hand Function Scale questionnaire, the Visual Analogue Scale, goniometry, a baseline pinch gauge, circumferential measurement, and the modified Kapandji Index. **Results**: There were significant improvements in the motor imagery group compared with the control group in post-motor imagery, pre- and post-rehabilitation measurements, functional pain (*p* < 0.001), rest pain (*p* < 0.01), hand mobility (*p* < 0.001), range of motion (*p* < 0.05), and wrist edema (*p* < 0.04); there were also improvements in pre- and post-rehabilitation measurements, quality of life in relation to upper limb function problems (*p* < 0.04), the post-rehabilitation measurement of hand functionality (*p* = 0.02), and post-motor imaging in finger-to-finger pinch strength. There were no statistically significant differences in the rest of the variables. **Conclusions**: Early intervention with motor imagery could be effective for resting and functional pain, quality of life in relation to upper limb problems, functional capacity, mobility, range of motion, strength, and edema.

## 1. Introduction

The thumb is an essential part of the body for carrying out daily living activities, participating in approximately 50% of our daily occupational performance [1]. However, when a diagnosis of trapeziometacarpal osteoarthritis is made, hand function is limited, which can be very disabling [2]. In addition, 1–7% of adults have pain in the trapeziometacarpal joint [3], resulting in structural alterations of the articular cartilage, subchondral bone, ligaments, capsule, synovial membrane, and periarticular muscles [4]. Approximately 22% of people over 50 years of age are symptomatic [5], it is very common in postmenopausal women [6], and there is a 6:1 incidence between women and men, a difference that decreases with increasing age [7].

In addition to physiological and biomechanical changes along with this symptomatology, cortical changes will occur [8], that will influence the planning, coordination and execution of fine hand movements, somatosensory changes, a modification of the body schema, and alterations in motor control [9,10]. When trapeziometacarpal osteoarthritis is established, conservative treatment is performed to delay the loss of functionality as much as possible, and in some cases, surgery must be considered; once surgery has been performed, the patient is immobilized with a cast or splint [11,12,13], which further deteriorates motor capacity with consequent cortical changes [14].

The scientific literature suggests that alterations in sensorimotor representation occur when the upper limb is immobilized [15,16,17], manifesting in proprioceptive alterations, deficits in right–left judgment, a decrease in speed in motor skills testing, and mental visualization, an altered body schema, deficits in manual dexterity [18], and changes in pain intensity and symptom duration [10].

To understand these neuronal modifications associated with chronic pathologies and limb immobilization, it is necessary to implement interventions that promote optimal recovery in rehabilitation services [19].

A considerable number of authors have proposed the use of motor imagery techniques in physical rehabilitation as a simple and low-cost tool to promote motor recovery and improve function [20,21,22,23,24], decrease pain [25], and improve motor performance [26].

Motor imagery is a mental simulation process that involves the systematic use of imagery and the mental rehearsal of a specific movement without actually executing it [27]. The most common MI modalities are the self-visualization of the movement and the ability to perceive the somatic feedback that the actual movement should elicit [28].

Several behavioral, brain imaging, and clinical studies have suggested that motor imagery (MI) and motor execution share similar characteristics and recruit largely overlapping brain networks and, so, its use is becoming more relevant in rehabilitation programs [29].

In recent years, numerous studies have been published regarding its usefulness in rehabilitation after central nervous system disorders [30,31] and for patients with upper limb injuries [32,33]. However, there have been very few clinical studies concerning the use of MI as a treatment during the immediate post-operative period following hand surgery [34].

In a recent and, to date, unique study on post-surgical motor imagery training in trapeziometacarpal joint osteoarthritis, Gandola et al. studied 22 patients with rhizarthrosis. Before and after surgery, patients underwent behavioral tests to measure pain and motor performance, and functional magnetic resonance imaging (fMRI) measurements of brain motor activity. After surgery, the affected hand was immobilized, and patients were enrolled in MI training. They found that those who complied with the scheduled motor imagery exercise program had increased brain activation, counteracting the adverse effects of immobilization, reducing pain, and expediting motor recovery [35].

The primary objective of this study was to analyze the efficacy of motor imagery training during the post-surgical immobilization period in women over 50 years of age who underwent surgery for trapeziometacarpal osteoarthritis, compared with the conventional protocol (surgery and immobilization with a plaster cast) in the baseline pre-surgery, post-surgery, pre-rehabilitation, and post-rehabilitation periods.

The secondary objectives were as follows: (1) to evaluate its effect on pain at rest and during activity; (2) to measure changes in range of motion (ROM); (3) to measure changes in digit–digital, tradigital, and lateral strength; (4) to evaluate its effect on wrist and hand edema; (5) to evaluate its effect on the functionality of activities of daily living; (6) to determine quality of life related to upper limb functionality; (7) to evaluate the clinical relevance of the variables of functional pain, pain at rest, quality of life in relation to the problems of the upper limb, and the functionality of the hand compared with the control group.

It was hypothesized that adding the motor imagery technique after surgery and during the three-week immobilization period would be more effective than the conventional protocol of surgery and immobilization in the rehabilitation of women over fifty years of age who have undergone surgery for trapeziometacarpal osteoarthritis and those who have been operated on for trapeziometacarpal osteoarthritis (TMC OA).

## 2. Materials and Methods

### 2.1. Study Design

An experimental, prospective, longitudinal, parallel-arm, superiority study, single-blind randomized clinical trial was conducted at León University Hospital. It was carried out in accordance with the CONSORT (Consolidated Standards of Reporting Trials) criteria.

The trial was approved by the Ethics Committee with the internal registration number 17155, and was registered in Clinical Trials (NCT03815734). Details of the trial design are described in the study protocol [36].

The start of this study was in 2018, but the end date (2023) could not be met according to the marked chronology, due to the COVID-19 pandemic situation, which caused the temporary suspension of non-urgent surgical interventions, and the trial was stopped.

#### 2.1.1. Participants

Participants were recruited at the León University Hospital. We asked permission from the Admissions Department for the surgical waiting list for plastic surgery. The plastic surgeon selected the patients with trapeziometacarpal osteoarthritis taking the exclusion and inclusion criteria of this study into account.

The occupational therapist contacted the selected patients by telephone to invite them to a face-to-face meeting where they were informed about the objectives and procedures of this study. Patients who voluntarily participated in this study signed an informed consent agreement, and were randomly assigned to one of the two study groups: control or experimental.

#### 2.1.2. Inclusion Criteria

Women over 50 years of age on the surgical waiting list at the University Hospital of León for surgery for trapeziometacarpal osteoarthritis and residents of the province of León were eligible for inclusion. They were operated on by plastic surgeons following four surgical techniques: arthroplasty with ARPE implant or trapeziectomy with interposition–suspension arthroplasty (techniques used: Zancolli, modified Weilby, Burton–Pellegrini) [12].

#### 2.1.3. Exclusion Criteria

Women with cognitive disorders that prevented them from following test commands and other injuries to the affected limb that interfered with the brain’s ability to create a mental image during MI were excluded from this study; patients with rheumatoid arthritis, trigger finger, de Quervain tenosynovitis, previous fractures, and carpal tunnel syndrome were also excluded.

### 2.2. Study Variables and Measurement Instruments

#### 2.2.1. Sociodemographic Variables

The initial data collection was carried out in the occupational therapy department by the principal investigator, and the data included age, employment, other hand pathologies, previous hand surgeries, dominant hand, and the hand awaiting surgery. Throughout the procedure, the following data were collected: date of pre-surgery, date of surgery, date of removal of post-surgical immobilization, and date of the start of protocolized rehabilitation.

#### 2.2.2. Clinical Variables

Functional and rest pain was measured using the Visual Analogue Scale (VAS), a validated instrument for the estimation of global pain on a scale of 0 to 10. This scale is suitable for use in patients with various types of osteoarthrosis. It consists of a single item comprising a horizontal line 10 cm (100 mm) in length and includes two verbal descriptors: ‘no pain’ (at 0 cm) and ‘pain as severe as possible’ (at 10 cm) [37].

The Disabilities of the Arm, Shoulder, and Hand (DASH) is a specific questionnaire widely used to assess health-related quality of life in patients with musculoskeletal disorders of the upper limb [38]. This instrument has been shown to be reliable, sensitive to change, and appropriate for use in the Spanish population. It asks questions about both physical and psychological aspects. It consists of 30 questions with scores from 1 to 5, and it is necessary to complete at least 27 of them to obtain a valid overall score. The values assigned to each question are added and divided by the number of questions answered, and this value is subtracted by 1 and multiplied by 25 to obtain a final result between 0 and 100 [39].

The Cochin Hand Functional Disability Scale (also known as the Duruöz Hand Index) was used to assess the participants’ ability to perform their activities of daily living (personal hygiene, dressing, and various other productive activities). It consists of 18 items, each with 6 possible score values from 0 to 5. An overall score of 0 represents the lowest level of disability, while the highest level of disability is indicated by a score of 90 [40].

Hand mobility was measured with the modified Kapandji Index, which has been validated to assess functionality and mobility of the hand using simple and easily reproducible measurements of wrist mobility, thumb opposition, and flexion–extension of the long fingers. It consists of 3 sub-indices with a possible score from 0 (worst mobility) to 100 (best mobility) [41].

The Moeltgen goniometer [42] was used to measure the active abduction ROM of the thumb. The intermetacarpal angle was assessed with the forearm in pronation, the wrist in neutral position, and the palm of the hand on a flat surface. The goniometer was placed at the carpometacarpal joint of the thumb, and both arms were placed on the first and second metacarpal heads in maximum radial abduction [43].

The strength that can be exerted before the onset of pain was measured using a Baseline pinch meter. Tip-to-tip pinch strength (DD) with the first and second fingers, lateral pinch (LT), and three-point pinch (TD) were measured. These measurements were performed with the patient seated, with the elbow at 90°, allowing one minute of rest time between tests. Each test was repeated three times, and measurements were recorded in kilograms [44].

Edema was assessed using circumferential measurement, taking bone references at the wrist level (radial and ulnar styloid) and the metacarpophalangeal joint. This perimetric measurement was carried out with a flexible tape measure.

A total of 4 measurements were taken over the 13 weeks of each participant’s process.

### 2.3. Interventions

#### Assessment

All outcome measures were administered by the clinical rehabilitation specialist, who was blinded to the assignment and treatment received. Four assessments were conducted throughout the intervention: 2 weeks before surgery (baseline measurement), 5 weeks after EI and after the implementation of MI training (post-imaging motor measurement), 7 weeks after EI and before the start of the standard rehabilitation protocol for both groups (pre-rehabilitation measurement), and 11 weeks after EI, at the end of the standard rehabilitation protocol, which consisted of paraffin baths, magnetotherapy, physical therapy, and/or conventional occupational therapy (post-rehabilitation measurement).

Before surgery, the occupational therapist conducted in-person motor imagery training for the group to explain the technique and provide instruction on the exercises they would perform during the 3-week immobilization period.

In addition, the occupational therapist instructed them to download the “laterality” application for the recognition of left- or right-hand images (laterality judgment), and to identify images of the right or left sides of the parts of the hand.

In addition, the Kinesthetic and Visual Imagination Questionnaire (KVIQ-20) was used to assess motor imaginative ability as a preparatory activity for the MI protocol that they had to perform.

Next, a 20-min session of action observation therapy was conducted with each participant, observing the movements and functional activities executed by the therapist, which were identical to those that, after surgery, they had to perform during post-surgical immobilization using motor imagery. In addition, a PowerPoint presentation with the MI exercises was sent to them as a reminder and support.

After surgery, all of the participants had to perform this treatment during the three-week period of post-surgical immobilization and, subsequently, the protocolized rehabilitation treatment.

Participants in the control group were immobilized for 3 weeks and subsequently underwent standard rehabilitation, which consisted of 15 sessions of magnetotherapy, paraffin baths, ROM exercises (passive and active), and strengthening exercises.

**Figure 1:**
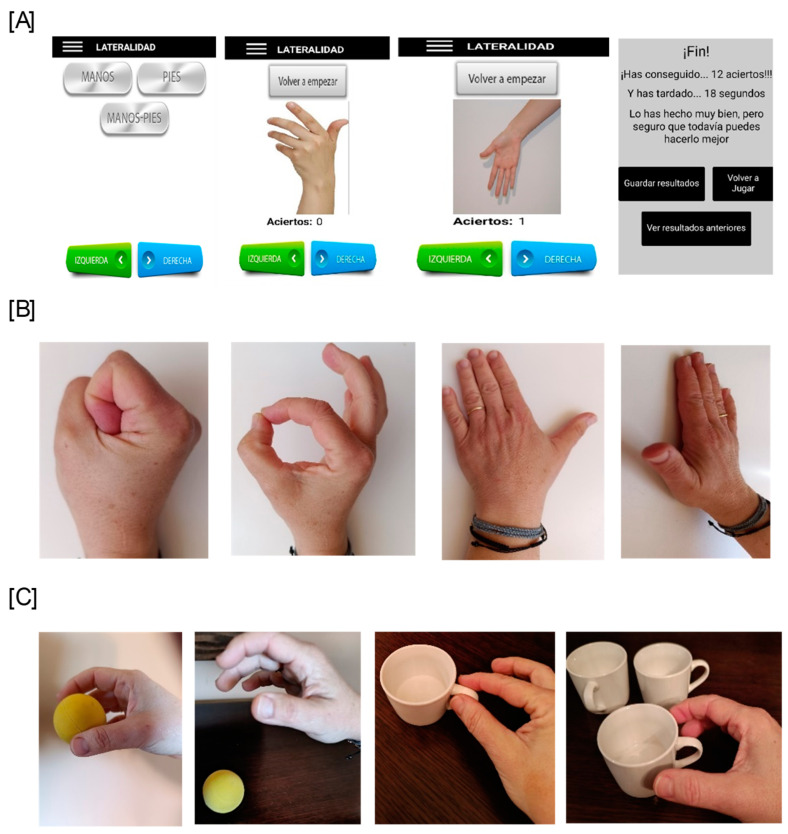
Exercises from the protocol followed by the motor imagery group. (**A**) Implicit MI. Laterality training. (**B**) Explicit MI. Internal hand visualization (abduction and extension, fist opening and closing, and finger opposition). (**C**) Explicit MI. Internal hand visualization (picking up a tennis ball from a table and dropping it, and picking up a coffee cup and placing it on a shelf).

Figure 1 shows the exercises performed by the participants in the motor imagery group.

- During the 3 weeks of postoperative immobilization, laterality training was performed twice daily (morning and evening) to assess the patients’ left–right discrimination (implicit MI).
- During the first two weeks of MI training, patients performed internal hand visualization, mentally repeating manual gestures (thumb abduction and extension, fist opening and closing, and finger opposition). These exercises were completed four times daily in 10-min training sessions with 4-h intervals (explicit MI). At the end of the second week, each participant was contacted by telephone to reinforce therapeutic adherence, to check that the participants performed the exercises, and to remind them of the exercises they were to add during the third week (explicit first-person motor imagery using two functional activities: picking up a tennis ball from a table and dropping it, and picking up a coffee cup and placing it on a shelf). During this phase, the total intervention time was increased to 15 min.

This protocol was carried out at home with favorable conditions for concentration in a seated or lying position and with eyes closed.

Throughout the 13 weeks for which the entire process lasted, participants had the opportunity to make phone calls to resolve any questions or incidents that might have arisen during the intervention and were called by the clinician in the Physical Medicine and Rehabilitation Center to carry out each measurement.

### 2.4. Sample Size

The calculation of the sample size was based on one of the few preceding studies whose intervention and criteria were similar to those of the present study [45], in which an RCT was carried out on 36 subjects with radius fracture (17 subjects in the experimental group, and 19 subjects in the control group) and DASH was considered to be the main variable.

At 8 weeks of treatment, with a mean (SD) of 43.90 (18.55) for the control group and 32.65 (12.95) for the experimental group, the statistical program G Power (Version 3.1.9.7, Düsseldorf University, Düsseldorf, Germany) was used to determine an effect size of d = 0.85, a level of α = 0.05, and a desired power of β = 80%.

With a ratio between the 2 groups (experimental and control) of 1 (N2/N1 = 1), a sample size of 18 patients per group was established. Therefore, considering a dropout rate of 10%, a total of 40 participants were recruited in this study.

### 2.5. Randomization

Concealed allocation was performed by a researcher unaware of participant recruitment and treatment using a computer-generated random number table (Random.org) and individual numbered cards stored in sealed opaque envelopes.

Before data collection and informed consent was signed, the therapist in charge of implementing the treatment opened the envelopes and assigned participants to either the motor imagery group or the control group.

### 2.6. Blinding

The rehabilitation physician conducted the participant assessments, and both the physician and the data analyst were blinded to the intervention group to which each subject was assigned. The therapist who delivered the intervention and the participants were not blinded.

### 2.7. Statistical Analysis

All statistical analyses were conducted using IBM SPSS Statistics version 27.0 (IBM Corp., Armonk, NY, USA). Descriptive statistics for continuous variables are reported as a mean ± standard deviation, while categorical variables are expressed as frequencies. The Shapiro–Wilk test was applied to evaluate the normality of the data distribution. Levene’s test was applied to check the homogeneity of variance, and Mauchly’s test was applied to evaluate the assumption of sphericity. When the sphericity assumption was not met, the Greenhouse–Geisser correction was used. To compare demographic characteristics between groups, the chi-squared test was used for categorical variables, and the *t*-test was applied for continuous variables. For group comparisons over time, an independent-samples *t*-test was performed when parametric assumptions were met, whereas the Mann–Whitney U-test was used when those assumptions were violated. A two-way repeated-measures analysis of variance (ANOVA) was employed to examine changes in dependent variables from baseline to post-rehabilitation assessments, considering group (experimental and control) and time (baseline, post-motor imagery, pre-rehabilitation, and post-rehabilitation). The interaction between time and group was also analyzed. To determine the effect size for the time × group interaction, partial eta squared (η^2^*p*) was calculated, with values of 0.01, 0.06, and 0.14 interpreted as small, moderate, and large effect sizes, respectively. For multiple comparisons, Cohen’s d was used to assess effect sizes, with thresholds of ≥0.2 for small, ≥0.5 for moderate, and ≥0.8 for large effects [46]. To assess the clinical relevance of statistically significant differences in the variables of functional pain, pain at rest, quality of life in relation to upper limb problems, and hand functionality, the odds ratio was calculated. The MCID for the VAS scale has been reported to be 20 mm [47]; for the DASH questionnaire, it has been reported to be 10.8 points [48], and for the Cochin questionnaire, it has been reported to be 3.4 points [49]. Statistical analysis was conducted at a 95% confidence level, and a *p*-value of less than 0.05 was considered statistically significant for all analyses.

### 2.8. Data Collection Methods

A data collection form was created, and each participant was provided with a document with the extension of the principal investigator and opening hours in case of any incidents.

The principal investigator was in charge of the computerized record of data and of any incidents that occurred during this study, such as adverse effects, abandonment of the study, losses due to refusal of surgery at the last moment, and so on. She was also in charge of preparing a quarterly report on the progress of this study for the ethics committee of the University Hospital of León, the Extremadura College of Occupational Therapists, and the University of León.

All personal data collected in the computerized form were linked to a number.

### 2.9. Ethical Considerations

All participants signed an informed consent form.

This clinical trial was approved by the Ethics Committee of the University Hospital of León with internal registration number 17155.

The principles set out in the 1964 World Medical Association Declaration of Helsinki and subsequent amendments, as well as the 1996 Council of Europe Convention on Human Rights and Biomedicine Regulation (EU) 2016/679 of the European Parliament and of the Council of 27 April 2016 on the protection of individuals with regard to the processing of personal data and on the free movement of such data, repealing Directive 95/46/EC (General Data Protection Regulation), were followed, in addition to Organic Law 3/2018 of 5 December on the Protection of Personal Data and the guarantee of digital rights, Law 41/2002 of 14 November, a basic law regulating patient autonomy and rights and obligations regarding clinical information and documentation, and Law 14/2007 of 3 July on Biomedical Research.

### 2.10. Funding

This study was funded by the College of Occupational Therapists of Extremadura (COPTOEX) in 2021. The funder played no role in the design of this study, data collection, analysis, or publication.

## 3. Results

**Figure 2:**
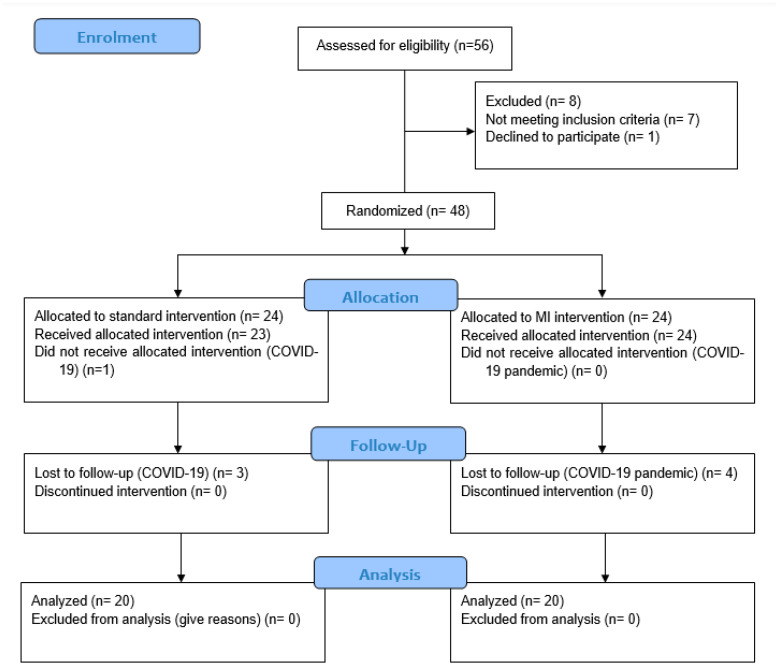
Flowchart.

Following the CONSORT standards, the following flowchart of the process results is shown (Figure 2).

The general characteristics of the participants are presented in Table 1. Fifty-six participants (n = 56) from the surgical waiting list were screened for eligibility criteria. Seven (n = 7) did not meet the inclusion criteria (five had carpal tunnel syndrome and two had a trigger finger), and one (n = 1) declined to participate. Finally, a total of 40 subjects (n = 40) participated in the current study. The age in the experimental group (62.65 ± 6.01) and in the control group (61.80 ± 5.52) did not differ significantly (*p* = 0.64). In occupation, type of surgery, dominant hand, and hand operated on, there were no significant differences between the groups.

**Figure 3:**
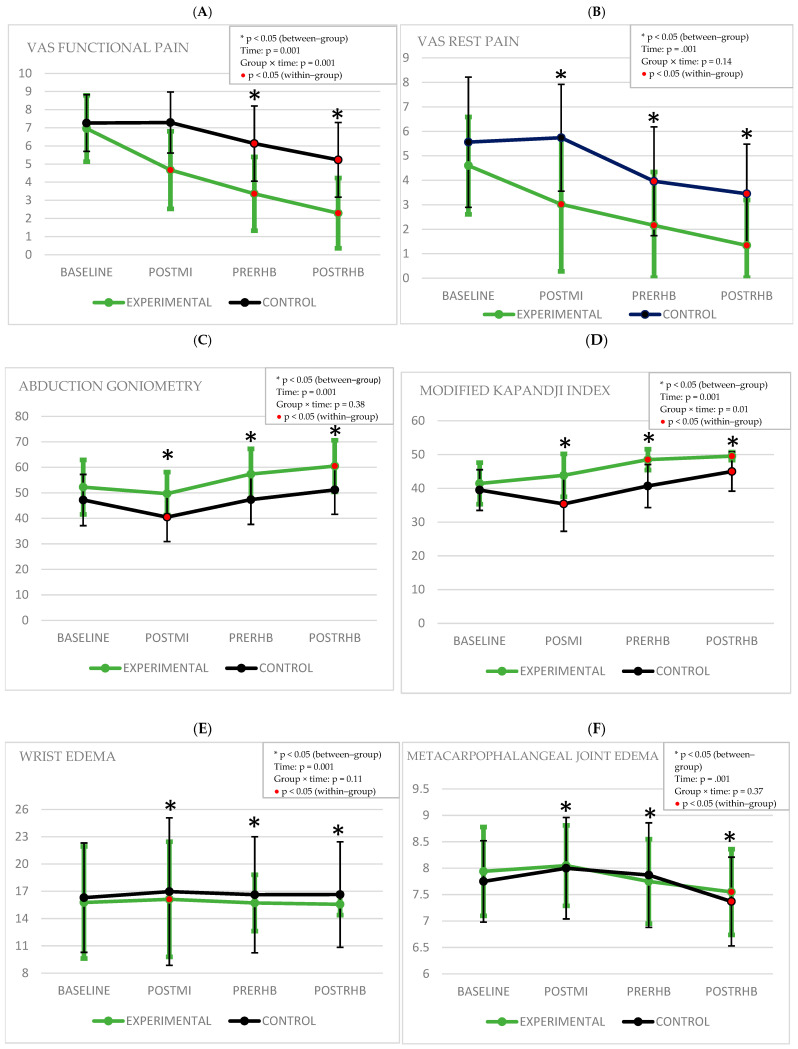

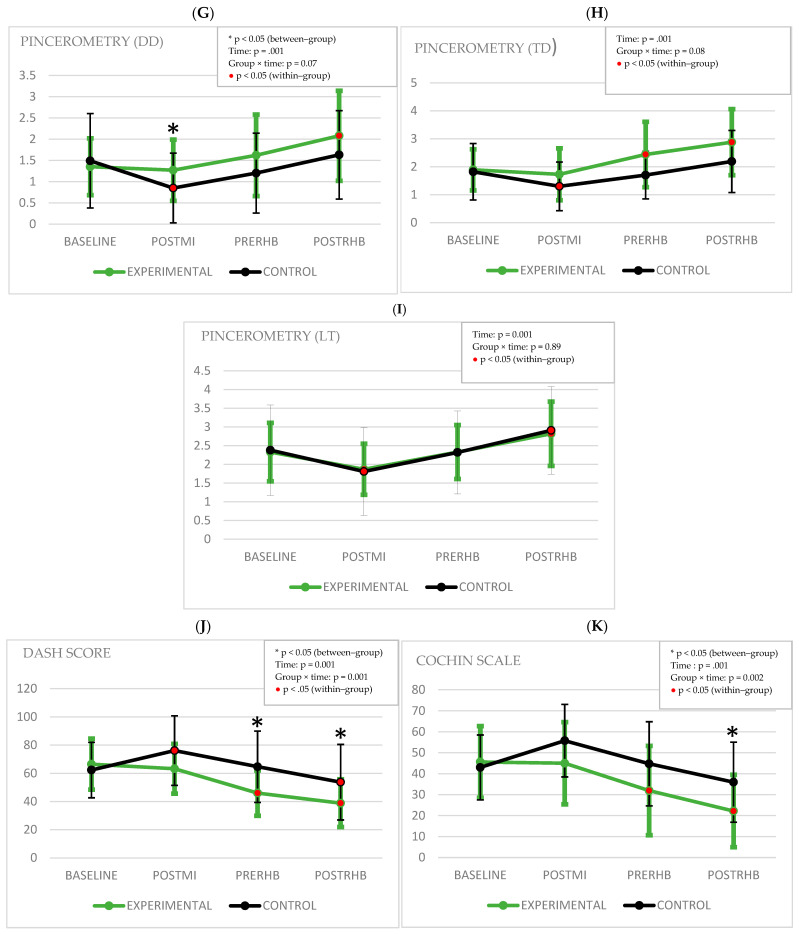
Graphical representation of the outcomes. (**A**) VAS for functional pain; (**B**) VAS for rest pain; (**C**) abduction goniometry; (**D**) Modified Kapandji Index; (**E**) wrist edema; (**F**) metacarpophalangeal joint edema; (**G**) pincerometry (tip-to-tip pinch); (**H**) pincerometry (lateral pinch): (**I**) pincerometry (three-point pinch); (**J**) Disabilities of the Arm, Shoulder, and Hand; (**K**) Cochin Hand Functional Disability Scale.

Comparisons of the outcome measurements within and between the groups are shown in Table 2 and Table 3, respectively. The MCID is shown in Table 4, and the graphical representation of the outcomes can be seen in Figure 3.

The two-way mixed-model ANOVA for functional pain (F = 7.97, *p* = 0.001, η^2^*p* = 0.17) revealed a significant interaction between group and time. Participants in the experimental group (EG) experienced greater reductions in functional pain compared to those in the control group (CG). Post hoc analysis in the EG demonstrated significant differences between baseline and post-motor imagery (postMI), baseline and pre-rehabilitation (preRHB), and baseline and post-rehabilitation (postRHB) (*p* < 0.001). By contrast, the CG showed no significant changes between baseline and postMI (*p* > 0.05), while significant differences were observed between baseline and both preRHB and postRHB (*p* < 0.001) (Table 2).

For rest pain, the two-way mixed-model ANOVA (F = 1.94, *p* = 0.14, η^2^*p* = 0.04) did not show a significant interaction between group and time. However, in the EG, post hoc analysis indicated significant reductions in pain between baseline and postMI, baseline and preRHB, and baseline and postRHB (*p* < 0.001). In the CG, no significant difference was found between baseline and postMI (*p* > 0.05), but significant changes were noted between baseline and both preRHB and postRHB (*p* < 0.001) (Table 2).

Between-group comparisons for both functional and resting pain revealed significant differences at postMI, preRHB, and postRHB (*p* < 0.05), with a large effect size favoring the EG (Table 3).

The two-way mixed-model ANOVA for goniometry (F = 0.92, *p* = 0.38, η^2^*p* = 0.25) did not indicate a significant group × time interaction. Post hoc analysis in the EG showed significant differences between baseline and postRHB (*p* < 0.05), whereas no significant differences were found between baseline and either postMI or preRHB (*p* > 0.05). In the CG, no significant changes were observed between baseline and preRHB or between baseline and postRHB (*p* > 0.05), but a significant difference was found between baseline and postMI (*p* < 0.05), attributed to a decrease in range of motion (Table 2).

In the between-group comparison, significant differences were observed in postMI, preRHB, and postRHB (*p* < 0.05), with a large effect size favoring the EG (Table 3).

For hand mobility, measured using the Kapandji Index, the two-way mixed-model ANOVA (F = 5, *p* = 0.01, η^2^*p* = 0.11) identified a significant group × time interaction. Participants in the EG showed greater improvements in hand mobility compared to those in the CG. Post hoc analysis in the EG indicated significant differences between baseline and both preRHB and postRHB (*p* < 0.001), whereas no significant changes were detected between baseline and postMI. By contrast, the CG showed no significant differences between baseline and preRHB (*p* > 0.05), while significant differences were observed between baseline and both postMI (reflecting a decline in hand mobility) and postRHB (*p* < 0.05), which were attributed to a reduction in range of motion (Table 2).

In the between-group comparison, there were statistically significant differences in postMI, preRHB, and postRHB measurements (*p* < 0.05), with a large effect size in favor of the EG (Table 3).

The two-way, mixed-model ANOVA of edema indicated no significant group × time interactions for either wrist edema (F = 2.26, *p* = 0.11, η^2^*p* = 0.08) or metacarpophalangeal joint edema. (F = 1.03, *p* = 0.37, η^2^*p* = 0.02). Post hoc analysis showed statistically significant differences for both the experimental and control groups between the baseline and postMI measurements (*p* > 0.05), but there was increased edema in both groups. Additionally, there were no statistically significant differences in either group between baseline and preRHB and between baseline and postRHB measurements (*p* > 0.05) (Table 2).

In the between-group comparison for both wrist edema and metacarpophalangeal joint edema, there were statistically significant differences in postMI, preRHB, and postRHB measurements (*p* < 0.05), with a large effect size in favor of the EG (Table 3).

The two-way, mixed-model ANOVA of pincerometry indicated no significant group × time interactions for DD pincerometry (F = 2.8, *p* = 0.07, η^2^*p* = 0.06), TD pincerometry (F = 2.44, *p* = 0.08, η^2^*p* = 0.06), or LT pincerometry (F = 0.12, *p* = 0.89, η^2^*p* = 0.003).

For DD pincerometry, post hoc analysis in the experimental group (EG) showed significant differences between baseline and post-rehabilitation (postRHB) (*p* < 0.05), whereas no significant differences were observed between baseline and either post-motor imagery (postMI) or pre-rehabilitation (preRHB) (*p* > 0.05). In the control group (CG), no significant differences were found between baseline and preRHB or between baseline and postRHB (*p* > 0.05), but a significant difference was noted between baseline and postMI (*p* < 0.05), which was associated with a decrease in strength.

For TD pincerometry, post hoc analysis in the EG revealed significant differences between baseline and both preRHB and postRHB (*p* < 0.05), while no significant differences were found between baseline and postMI (*p* > 0.05). In the CG, no significant differences were detected between baseline and either preRHB or postRHB (*p* > 0.05), but a significant reduction in strength was observed between baseline and postMI (*p* < 0.05).

Regarding LT pincerometry, post hoc analysis indicated significant differences between baseline and postMI (due to a reduction in strength) and between baseline and postRHB (*p* > 0.05) in both groups. Additionally, neither the EG nor the CG showed significant differences between baseline and preRHB (*p* > 0.05) (Table 2).

In the between-group comparison, no significant differences were found in postMI, preRHB, and postRHB measurements (*p* > 0.05), except for the postMI measurement in DD pincerometry, where a significant difference was observed (*p* < 0.05) (Table 3).

The two-way mixed-model ANOVA for DASH scores (F = 8.74, *p* = 0.001, η^2^*p* = 0.18) showed a significant interaction between group and time. Participants in the EG demonstrated greater improvements in DASH scores compared to those in the CG. Post hoc analysis in the EG revealed significant differences between baseline and both preRHB and postRHB (*p* < 0.001), while no significant differences were observed between baseline and postMI (*p* > 0.05). In the CG, no significant changes were detected between baseline and preRHB (*p* > 0.05), but significant differences were found between baseline and both postMI (attributed to an increase in disability) and postRHB (*p* < 0.001) (Table 2).

In the between-group comparison, significant differences were observed at postMI, preRHB, and postRHB (*p* < 0.05), with a large effect size favoring the EG (Table 3).

For Cochin scores, the two-way mixed-model ANOVA (F = 6.31, *p* = 0.002, η^2^*p* = 0.14) indicated a significant interaction between group and time, with the EG showing greater improvements than the CG. In the EG, post hoc analysis revealed significant differences between baseline and both preRHB and postRHB (*p* < 0.001), while no significant differences were detected between baseline and postMI (*p* > 0.05). By contrast, the CG showed no significant differences between baseline and preRHB or between baseline and postRHB (*p* > 0.05), but significant differences were observed between baseline and postMI, which were attributed to a decline in functionality (Table 2).

Between-group comparisons revealed significant differences in postMI, preRHB, and postRHB measurements (*p* < 0.05), with a large effect size in favor of the EG (Table 3).

The MCID analysis based on the difference in scores between baseline and postMI measurements and between baseline and postRHB measurements, as well as the calculation of the OR for the self-reported variables of functional pain, pain at rest, DASH, and Cochin, is presented in Table 4.

Patients who received treatment with MI were more likely to exceed the MCID in functional pain at the postMI measurement (OR = 16.7; 95% CI: 2.97–93.88; *p* < 0.001) and at the postRHB measurement (OR = 4; 95% CI: 0.98–16.27; *p* < 0.001), DASH at the postRHB measurement (OR = 7.4; 95% CI: 1.77–31.04; *p* < 0.001), and Cochin at the postRHB measurement (OR = 9; 95% CI: 1.63–49.44; *p* < 0.001). However, no significant differences were observed in resting pain at the postMI measurement (OR = 1.55; 95% CI: 0.42–5.76; *p* > 0.05) or at the postRHB measurement (OR = 2.27; 95% CI: 0.63–8.1; *p* > 0.05), in DASH at the postMI measurement (OR = 2.2; 95% CI: 0.36–13.97; *p* > 0.05), and in Cochin at the postMI measurement (OR = 2.1; 95% CI: 0.51–9; *p* > 0.05).

## 4. Discussion

In this study, the efficacy of motor imagery training applied early during the period of immobilization with a plaster cast was tested for women over 50 years of age who underwent surgery for trapeziometacarpal osteoarthritis.

Our study has several points of interest. In particular, it is a novel study due to the scarce literature on the intervention of motor imagery in the acute period after both upper and lower limb surgeries. This is the second published study on the early application of motor imagery in trapeziometacarpal osteoarthritis. The sample was collected while taking the perspective of gender into account, considering the high prevalence in women—35.8% in women [50] compared with 7% in men [51]—so 100% of the participants in our study were women. This was a preliminary step to be able to analyze the causes of differences in outcomes between men and women and the differences in demographics in subsequent studies, providing a better understanding of the overall health status of this condition. This is an innovative, easy-to-apply intervention with no side effects, and it is cost-effective. In our study, we also assessed the potential benefit in our daily clinical practice by measuring patient-rated outcomes and analyzing the minimum clinically important difference.

Patients reported persistent pain at the base of the thumb, which limited hand functionality [52] and thumb mobility [53], and affected the performance of activities of daily living that required precision and grip strength, making work, home, and self-care activities substantially more difficult as the disease progressed [54]. These changes could trigger a decrease in the neural networks manifesting impaired planning, coordination, and execution of fine movements, as well as for tactile acuity, body image, and motor control. In addition, patients undergo surgery followed by a period of immobilization and disuse of the hand, which may induce cortical changes and decreased motor performance [55]; therefore, it is at this precise moment that motor imagery could be a novel potential treatment option when there is limited applicability of direct physical training [56].

Motor imagery has been used in the acquisition of new motor gestures, improved motor performance, and improvements in the range of motion [57]; it is also used for the treatment of pain [58] and in neurological pathology [30], but few authors have focused on early motor imagery treatment in traumatic pathology after surgery. Stenekes et al. [59] analyzed changes after flexor tendon surgery, Dilek et al. analyzed them after distal radius fracture [45], and Guillot et al. analyzed them in the treatment of hand burns [60]. Gandola et al. [35] published a study in 2019 in which they tested the hypothesis that motor imagery training could mitigate the consequences of hand immobilization after surgical treatment of rhizarthrosis, and that this improvement would be accompanied by significant changes in brain activity based on the principles of brain plasticity. Other studies have immobilized the upper limbs in healthy people to analyze the effectiveness of MI treatment [20,61].

In our study, prior to applying the intervention, the imagery ability of the participants in the experimental group was measured according to their ability to visualize and feel imagined movements using the Kinesthetic and Visual Imagery Questionnaire (KVIQ). Consistent with other studies [62,63], the participants obtained better visual than kinesthetic scores. Three reported difficulties in visualizing mentally, although, with practice, these difficulties disappeared.

In terms of pain, it should be remembered that the most common cause of chronic pain in the world is osteoarthritis, with significant adverse consequences in various areas of a patient’s life [64]. In addition to the pain of TMC OA itself, acute postoperative pain must be taken into account after surgery, without forgetting that both the surgery and the experience of pain can be influenced by multiple factors. In the present clinical trial, the participants underwent either interposition–suspension arthroplasty or prosthetic arthroplasty. It is interesting to note when discussing the results that multiple studies have analyzed comparisons of postoperative pain scores between the two surgeries, and most found a clinically insignificant difference in postoperative pain outcomes between the two procedures [65,66], although the type of operation did not differ significantly between the groups in our study.

### 4.1. Pain

Our findings on the participants’ perception of both pain at rest and pain with movement, with statistically significant intergroup differences in all three measurements after surgery and a high effect size, show that MI is effective in reducing pain both functionally and at rest in patients with rhizarthrosis. Of note is the post-immobilization/motor imagery measurement (5 weeks after surgery) in the MI group (implicit and explicit first-person MI) as the pain intensity decreased; while in the control group, it slightly increased, suggesting that motor imagery training produces clinical improvement after cast removal.

The mechanisms that could explain this improvement are still being studied. Considering the chronic nature of rhizarthrosis, and that evidence has shown that immobilization and disuse produce plastic brain changes at both the motor and perceptual or somatosensory levels [67], one hypothesis would be that proposed by Moseley, who states that the sequential activation of premotor and motor cortical regions by means of MI without evoking pain could reduce it [68]; or there may be a mechanism capable of reversing the maladaptive neuroplastic changes that occur in TMC OA due to its chronicity and the consequences of post-surgical immobilization, as argued by de Souza et al. in 2015. In particular, they hypothesized three different processes: the release of enkephalin and meta-enkephalin in analogy with physical exercise, the modulation of pain perception at the spinal level (dorsal horn), and the brain neuromodulatory process leading to the inhibition of the pain pathway [69].

Gandola et al. added the possibility of an analgesic effect of MI training on pain mediated by cortical plasticity processes induced by simulation and repetitive motor trials [35].

Our results are consistent with those of Gandola et al. who, in their study, applied MI to rhizarthrosis patients during 2 weeks of splinting, revealing that motor imagery training was sufficient to counteract the negative effects of hand immobilization after surgery by reducing the magnitude of pain [35].

These observations could confirm the clinical potential of the early application of the MI technique for pain, as previously reported in other studies in which there were statistically significant differences, such as the study of Dilek et al., who performed MI during the immobilization period after wrist fracture surgery, and in those of Moukarzel et al. [70] and Zapparoli et al., who measured acute postoperative recovery in patients undergoing total knee arthroplasty [71], observing a significant decrease in pain, but not in the study by Stenekes et al. [59], where no significant changes were found between groups after flexor tendon surgery.

Previous studies have also demonstrated the efficacy of different techniques based on motor imagery for the treatment of pain in musculoskeletal disorders [72,73]. In recent systematic reviews and meta-analyses, such as that of Yap and Lim, who, in 2019, analyzed the effects of motor imagery in musculoskeletal disorders [74]; that of Galonski et al., who measured the possible benefits for knee pain in 2023 [75]; that of Limakatso et al., who addressed phantom limb pain [76]; and that of Ríos-León et al., who addressed complex regional pain syndrome in 2024 [77]. While it was generally considered that the motor imagery technique could be beneficial, the existing studies had small sample sizes and there remains a need for future research of high methodological quality.

### 4.2. Range of Motion and Mobility

With regard to the joint range in the abduction of the first finger measured in this study with goniometry, the opposition of the thumb to the triphalangeal fingers, and the support of the hand in plane measured with the Kapandji test, the statistically significant differences obtained in the three measurements after surgery and immobilization and the large effect size—except in the post-rehabilitation measurement in the goniometry variable—confirmed that the motor imagery treatment was effective. The post-motor imagery measurement (5 weeks after surgery and immobilization) was noteworthy, as the MI group had a less pronounced drop in mobility than the control group in abduction; it not only maintained baseline mobility, but there was a slight increase in opposition and flat support, so we can argue that motor imagery had a protective effect on the consequences of immobilization after surgery.

In this study, improvement in pain and joint range could be effective in facilitating the initiation of light manipulative activities, thus promoting autonomy in self-care and preventing altered movement patterns when initiating activities of daily living, as well as arriving at standard occupational therapy and physiotherapy rehabilitation with reduced maladaptive behaviors or movements as a learned or conditioned response to pain with movement.

Movement commonly evokes pain in people with chronic painful conditions, presumably because movement activates nociceptors [78], and patients often have a fear of movement [79]. When surgery is performed and the cast is removed, movement limitation occurs and may be accompanied by kinesiophobia as a protective mechanism; in addition, according to the literature, three other factors influencing a joints’ range of motion can be deduced; namely, structural and biomechanical factors (i.e., ligaments and capsules around the joints, muscles, and tendons and possibly edema) [80] that could be derived from the surgical intervention; somatosensory factors (i.e., proprioception and kinesthesia) [81], and motor control at the cortical level [82]—which, in this case, could have been shaped or modulated by motor imagery. This was confirmed by a recent systematic review and meta-analysis which concluded that the use of MI may be beneficial in groups with musculoskeletal injuries in which pain and reduced range of motion are prevalent [74].

### 4.3. Edema

In our study, we consider it vitally important to intervene in acute–subacute inflammatory edema early to avoid later complications, such as complex regional pain syndrome, decreased oxygen perfusion that hinders healing, and progressive fixation of collagen fibers in ligaments and tendons causing fibrosis, joint stiffness and, consequently, a vicious cycle of decreased mobility, fear of movement, and loss of autonomy [83]. For this reason, it was hypothesized that early motor imagery treatment could improve the patients’ edema measured at the wrist and metacarpophalangeal joints. The results provided us with striking data. Firstly, at the wrist level, the significant statistical differences in the pre- and post-rehabilitation post-motor imagery measurement and large effect size led us to conjecture that the hypothesis was fulfilled; secondly, it could not be explained why the edema, after conventional rehabilitation, slightly increased at the wrist level in the experimental group, and yet improved at the metacarpophalangeal level.

The scientific literature on motor imagery treatment for hand edema is very scarce. Neurophysiological aspects such as the modulation of corticospinal excitability and the involvement of the autonomic nervous system could be related to changes following motor imagery treatment [84]. Moseley et al. further argued that symptomatology could be modulated by beliefs about pain and movement that could influence inflammation in patients with chronic upper limb pathologies [78]. Performing a motor imagery treatment in complex regional pain syndrome, Moseley observed a reduction in edema [68], and proposed the sequential activation of cortical motor networks, starting with implicit motor imagery and continuing with explicit imagery and, finally, mirror therapy—the latter of which could not be followed in this study due to the bilateral nature of trapeziometacarpal osteoarthritis [72].

### 4.4. Strength

In terms of digit–digital, tradigital, and lateral strength, starting from the baseline measurement with no significant statistical differences, the post-imaging motor measurement (5 weeks after surgery and immobilization) stands out, as we observed a more marked decrease in strength in the control group. Thus, it seems that the imagery treatment had a protective effect, attenuating the losses caused after surgery, especially in digit–digital strength, where there were significant differences, although with a small effect size. It can be conjectured that this early improvement favored the recovery of strength throughout the intervention process, except in lateral pincerometry, where the difference at the end of the intervention was insignificant between the groups.

In 2023, Liu et al. [85] published a systematic review and meta-analysis that aimed to determine the effectiveness of motor imagery training in improving maximal voluntary muscle contraction strength for healthy young and older adults; they concluded that the benefit was mainly in its application to small muscles in older people, and it could be considered in rehabilitation settings when physical training is too demanding for patients with motor disabilities, cannot be performed, or is conducted in patients who are susceptible to injury.

Paravlic et al. [86] published a systematic review and meta-analysis in 2018 on the effects of the motor imagery technique in healthy subjects, finding improvements ranging from 5% to 30% in the 13 included studies, and suggesting that various forms of MI practice have the potential to improve maximal muscle strength.

In studies in which early upper limb motor imagery treatment has been carried out—as in our study, those in healthy people who had their upper limbs immobilized [20,87], and those in people with trauma pathologies [45,59]—the results have been mixed, but all highlight the beneficial effects of the therapy in preventing the loss of strength associated with short-term immobilization.

The resulting strength gains from motor imagery could involve improved representation of force generation and enhanced cortical planning, promoting an adaptive neuroplastic process; subsequently, this may influence motor recruitment and motor unit synchronization at the peripheral level [70,88]. These mechanisms would contribute to increased neural impulse output to agonist muscles [89] and inhibit antagonist muscle activation, leading to improved fiber synchronization and increased muscle activity [90], thereby improving strength [91].

### 4.5. Quality of Life in Relation to Upper Limb Problems Dependent on Hand Function and Occupational Performance and Functionality

This study provides interesting findings regarding the participants’ perception of their quality of life in relation to upper limb problems dependent on hand function and occupational performance, as measured with the DASH questionnaire and the Cochin Hand Functional Disability Scale (CHFS). Perceived disability and global upper limb symptoms, including physical, social, and psychological aspects and activities of daily living in the domain of personal hygiene, dressing, and certain productive activities, were addressed. First, the baseline measures of DASH (64.45 out of 100%) and CHFS (44.3 out of 90 points) indicated high perceived disability and loss of function. These results were consistent with those of Gandola et al., who conducted a study in which they concluded that the presence of rhizarthrosis without surgery already had a significant impact on the ability to perform daily activities with the affected hand [8]. Furthermore, with surgery and 3 weeks of immobilization, there would be negative consequences for functionality and occupational performance; this is why, in the present study, we attempted to check whether MI treatment would have benefits. In the results obtained, on one hand, the significant differences between the groups occurred in the final part of the intervention with a medium effect size, perhaps as a consequence of the improvements obtained previously in the post-motor imagery measurement, especially in functional and rest pain, joint range, and mobility; in addition, there were lower losses after surgery in the MI group, which favored a greater recovery of functionality at the end of rehabilitation. These early improvements may also have influenced cognitive aspects, such as by causing less fear of movement and greater self-confidence in gradually integrating the operated hand into activities of daily living more easily. On the other hand, after surgery and MI treatment and during immobilization, the experimental group improved their autonomy, while the control group had losses, so we can conclude that the MI treatment had a protective effect against the possible loss of function that may be caused by post-surgery immobilization.

In the DASH questionnaire [39], a score equal to or greater than 50 points is considered to produce a perception of the presence of difficulties in daily occupational performance in subjects, which occurred with our control group participants at the end of the final assessment, who did not achieve a score lower than 50 points (53.76) after performing conventional rehabilitation; however, the participants who performed MI achieved lower scores (38.87), as they were able to interpret adequate functioning in their daily activities despite certain difficulties that may occur.

These observations extend the findings from previous studies that demonstrated the efficacy of MI for functional capacity [33,45], indicating a mechanism of activation and organization of cortical motor networks that would improve function and disability [92].

### 4.6. Minimum Clinically Important Difference

Finally, we wanted to highlight the importance of the observed clinical improvements, as the changes in the different health conditions usually evaluated in clinical practice and research require interpretation beyond their statistical significance. For this reason, in this study, in addition to this analysis, we considered the assessments made from the perspective of the participants in relation to the treatment and their state of health, obtaining self-assessed results that indicated changes and the progress of their recovery, and linking them to clinical decision making in rehabilitation.

Based on the previous reports indicating that patients are more interested in their ability to complete daily and functional activities [93] than in their pain [94], in addition to the statistical analysis of the variables under study, an analysis was performed while considering the minimum clinically important difference (MCID) for the VAS, DASH, and CHFS scales. The MCID is defined as the smallest clinical change that is important to a patient [47].

In functional and rest pain, especially pain in movement, more participants in the experimental group reached the MCID in the post-motor imagery and post-rehabilitation measurements than in the control group. The same was true for health-related quality of life and upper limb functionality measured with the DASH and CHFS.

Therefore, these findings support this rehabilitative approach to patient care and management in the early stages after surgery, as patients are more likely to show relevant changes if they have undergone MI treatment.

In the literature that was reviewed, no other articles where motor imagery treatment was carried out assessed the MCID.

### 4.7. Limitations

It is interesting to note that there are a limited number of experimental trials on the early application of motor imagery, and further research is required to obtain more definitive results and more solid conclusions regarding the efficacy of this technique; thus, the results of the present study and their interpretation should be treated with caution.

This study was not conducted with a previously established protocol, so rigorous research would also be needed to optimize training strategies and protocols to guide this approach.

Another aspect to consider in future research would be to assess recovery times, in order to determine the benefits in terms of medication use, return to work, and cost reduction.

It remains to be investigated whether motor imagery training in combination with other movement representation techniques, such as mirror therapy and action observation techniques, could improve outcomes.

Another limitation of this study is that the sample consisted exclusively of women over 50 years of age, which limits the generalizability of the results to other populations, such as men or younger individuals. Hormonal, biomechanical, and pain perception differences may influence functional recovery after rhizarthrosis surgery; the results obtained may not be applicable to these other groups. The age of the participants could have influenced their response to treatment, particularly in terms of neuroplasticity and motor learning. In younger populations, the effects of the intervention may differ.

The findings obtained in the present study could have promising clinical implications with applications in a wide pathological repertoire, potentially addressing questions that remain open through future research.

The findings obtained in the present study could have promising clinical implications with applications in a wide pathological repertoire in hand pathology where surgery and immobilization or immobilization alone is required, thus initiating early rehabilitation.

All these questions remain open through future research.

## 5. Conclusions

This is the first RCT to analyze the effects of a motor imagery intervention in the immediate postoperative period in women over 50 years of age who were operated on for trapeziometacarpal osteoarthritis on pain, upper limb function-related quality of life, function, mobility, joint range, strength, and edema.

Patients who received motor imagery treatment during the postoperative immobilization period showed a significant reduction in functional and resting pain compared to the control group, in terms of quality of life related to upper limb function, both before and after rehabilitation treatment, suggesting a considerable improvement in the functional perception of the affected limb, including the physical, social, and psychological dimensions.

Functionality and autonomy for the activities of daily living were significantly better for the experimental group after completion of rehabilitation, reflecting greater recovery of functional capacity compared to the control group.

In terms of hand mobility, the intervention group showed significant improvements in thumb movement capacity, suggesting a notable increase in joint range of motion.

Motor imagery could have a specific positive impact on certain types of hand grip strength and localized edema. The differences observed between the groups for functional pain, pain at rest, upper limb-related quality of life, and hand function exceeded the minimum clinically important difference (MCID).

Our results suggested that the motor imagery technique performed during the immobilization period after trapeziometacarpal osteoarthritis surgery seems to be effective in the short- and medium-term, and could contribute to the prevention of physiological side effects and the improvement of general well-being through reducing pain and functional losses while favoring better performance in activities of daily living. Our results also underline the clinical relevance of motor imagery interventions versus the standard treatment.

The inclusion of motor imagery in motor rehabilitation programs is emerging as a beneficial approach to functional recovery, especially in contexts where physical practice is limited or not possible. Its early implementation could promote a novel approach, which is as yet underexplored in the context of physiotherapy and occupational therapy, thus broadening the intervention strategies available to improve rehabilitation outcomes.

## Figures and Tables

**Table 1:** General characteristics of the participants.

Variables	EG	CG	X^2^/t
Age (Mean ± SD)	62.65 (6.01)	61.80 (5.52)	0.64
Employment Situation (A/R)	10/10	10/10	1.00
Surgery (AR/TP)	5/15	6/14	0.72
Dominant hand (R/L)	19 /1	18 /2	0.54
Operated hand (R/L)	10 /10	14 /6	0.33

SD: standard deviation; A: active; R: retired; AR: arthroplasty Arpe; TP: trapeziectomy; with interposition–suspension arthroplasty; R: right; L: left; EG: experimental group; CG: control group.

**Table 2:** Analysis of variance for all outcome measurements.

Variable		Difference Base−postMI (95% CI)	Difference Base−preRHB (95% CI)	Difference Base−postRHB (95% CI)	Time F (*p*)	Time × Group	
						F (*p*)	η^2^*p*
**VAS FP**	EG	2.28 * (1.34\3.22)	3.59 * (2.6\4.58)	4.67 * (3.47\5.68)	44.07 (0.001)	7.97 (0.001)	0.17
	CG	−0.03 (−0.97\0.91)	1.13 * (0.14\2.12)	2.03 * (0.83\3.23)			
**VAS RP**	EG	1.58 * (0.25\2.92)	2.44 * (1.15\3.73)	3.26 * (0.79\2.56)	21.52 (0.001)	1.94 (0.14)	0.04
	CG	−0.18 (−1.51\1.15)	1.60 * (0.31\2.88)	2.10 * (0.90\3.31)			
**GONIOMETRY**	EG	2.55 (−2.56\7.66)	−5.1 (−10.63\0.43)	−8.25 * (−14.1\−2.42)	15.7 (0.001)	0.92 (0.38)	0.25
	CG	6.7 * (1.58\11.81)	−0.15 (−5.68\5.38)	−4 (−9.82\1.82)			
**KAPANDJI**	EG	−2.4 (−6.27\1.47)	−7.05 * (−10.2\−3.88)	−8.1 * (−10.66\−5.33)	28.24 (0.001)	5 (0.01)	0.11
	CG	4.1 * 80.22\7.97)	−1.2 (−4.36\1.96)	−5.5 * (−8.06\−2.93)			
**EDEMA** (**WRIST**)	EG	−0.34 * (−6.55\−0.35)	0.05 (−0.31\0.41)	0.2 (−0.2\0.6)	9.06 (0.001)	2.26 (0.11)	0.08
	CG	−0.67 * (−0.98\−0.36)	−0.32 (−0.68\0.35)	−0.34 (−0.74\0.06)			
**EDEMA** (**MCF**)	EG	−0.1 (−0.4\0.18)	0.19 (−0.14\0.52)	0.39 * (0.1\0.68)	10.87 (0.001)	1.03 (0.37)	0.02
	CG	−0.25 (−0.54\0.04)	−0.12 (−0.460.21)	0.38 * (0.08\0.67)			
**PINCEROMETRY** (**DD**)	EG	0.11 (−0.26\0.49)	−0.27 (−0.7\0.16)	−0.73 * (−1.18\−0.28)	15.4 (0.001)	2.8 (0.07)	0.06
	CG	0.63 * (0.26\1.01)	0.29 (−0.14\0.72)	−0.13 (−0.58\0.31)			
**PINCEROMETRY** (**TD**)	EG	0.16 (−0.24\0.57)	−0.54 * (−1.01\−0.07)	−0.98 * (−1.48\−0.48)	18.76 (0.001)	2.44 (0.08)	0.06
	CG	0.52 * (0.11\0.92)	0.12 (−0.34\0.59	−0.36 (−0.86\0.13)			
**PINCEROMETRY** (**LT**)	EG	0.46 * (0.04\0.88)	0.002 (−0.35\0.47)	−0.48 * (−0.93\−0.03)	21.48 (0.001)	0.128 (0.89)	0.003
	CG	0.57 * (0.15\0.99)	0.006 −0.35\0.47)	−0.52 * (−0.97\−0.07)			
**DASH**	EG	3.26 (−4.28\10.81)	20.52 * (12.42\28.61)	27.66 * (19.42\35.9)	36.32 (0.001)	8.74 (0.001)	0.18
	CG	−13.82 * (−21.37\−6.26)	−2.33 (−10.43\5.75)	8.61 * (0.36\16.85)			
**COCHIN**	EG	0.6 (−5.45\6.65)	13.65 * (6.56\20.73)	23.4 * (15.41\31.39)	35.74 (0.001)	6.31 (0.002)	0.14
	CG	−12.8 * (−18.85\−6.75)	−1.75 −8.83\5.32)	7.02 (−0.97\15.01)			

*: *p* < 0.05; base: baseline; postMI: post-motor imagery; preRHB: pre-rehabilitation; postRHB: post-rehabilitation; CI: confidence interval; DD: tip-to-tip pinch; LT: lateral pinch; TD: three-point pinch; VAS: Visual Analogue Scale; DASH: Disabilities of the Arm, Shoulder, and Hand; FP: functional pain; RP: rest pain; MCF: metacarpophalangeal; EG: experimental group; CG: control group.

**Table 3:** Comparison of outcome measures between groups.

Variable	GROUP (Mean ± SD)		*p* Value	Effect Size
	EG	CG		
**VASFP**				
Baseline	6.96 (1.83)	7.26 (1.56)	0.41	
PostMI	4.67 (2.14)	7.29 (1.68)	0.001	1.36
PreRHB	3.36 (2.03)	6.13 (2.07)	0.0001	1.34
PostRHB	2.29 (1.94)	5.23 (2.06)	0.001	1.46
**VASRP**				
Baseline	4.60 (1.99)	5.56 (2.66)	0.2	
PostMI	3.02 (2.74)	5.74 (2.18)	0.002	1.01
PreRHB	2.16 (2.18)	3.96 (2.22)	0.013	0.81
PostRHB	1.34 (1.86)	3.45 (2.03)	0.002	0.92
**GONIOMETRY**				
Baseline	52.25 (10.69)	47.20 (10.05)	0.132	
PostMI	49.70 (8.47)	40.50 (9.58)	0.003	1.01
PreRHB	57.35 (9.89)	47.35 (9.70)	0.003	1.02
PostRHB	60.50 (10.11)	51.20 (9.60)	0.005	0.23
**KAPANDJI**				
Baseline	41.45 (6.17)	39.5 (6.02)	0.31	
PostMI	43.85 (6.34)	35.4 (8.11)	0.001	1.16
PreRHB	48.5 (3.10)	40.7 (6.39)	0.001	1.55
PostRHB	49.55 (1.19)	45 (5.81)	0.001	1.18
**EDEMA** (**WRIST**)				
Baseline	15.77 (6.17)	16.3 (6.02)	0.17	
PostMI	16.12 (6.34)	16.97 (8.11)	0.04	0.88
PreRHB	15.72 (3.10)	16.62 (6.39)	0.001	0.92
PostRHB	15.57 (1.19)	16.64 (5.81)	0.001	0.98
**EDEMA** (**MCP**)				
Baseline	7.94 (0.85)	7.75 (0.77)	0.45	
PostMI	8.05 (0.76)	8 (0.96)	0.08	0.06
PreRHB	7.75 (0.80)	7.87 (0.99)	0.67	0.13
PostRHB	7.55 (0.81)	7.37 (0.84)	0.49	0.21
**PINCEROMETRY** (**DD**)				
Baseline	1.35 (0.67)	1.49 (1.11)	0.82	
PostMI	1.27 (0.72)	0.85 (0.82)	0.030	0.48
PreRHB	1.62 (0.96)	1.20 (0.94)	0.11	0.44
PostRHB	2.08 (1.06)	1.63 (1.04)	0.18	0.43
**PINCEROMETRY** (**TD**)				
Baseline	1.89 (0.74)	1.82 (1.01)	0.81	
PostMI	1.73 (0.93)	1.30 (0.87)	0.14	0.47
PreRHB	2.44 (1.17)	1.70 (0.85)	0.08	0.71
PostRHB	2.88 (1.18)	2.19 (1.11)	0.06	0.60
**PINCEROMETRY** (**LT**)				
Baseline	2.33 (0.78)	2.38 (1.21)	0.87	
PostMI	1.87 (0.68)	1.81 (1.17)	0.85	0.06
PreRHB	2.33 (0.72)	2.32 (1.11)	0.98	0.01
PostRHB	2.82 (0.86)	2.91 (1.17)	0.78	0.08
**DASH**				
Baseline	66.53 (18.12)	62.37 (19.69)	0.57	
PostMI	63.26 (17.61)	76.19 (24.66)	0.06	0.60
PreRHB	46.01 (16.11)	64.71 (25.30)	0.008	0.88
PostRHB	38.87 (16.91)	53.76 (26.81)	0.04	0.66
**COCHIN**				
Baseline	45.60 (17.11)	43.00 (15.49)	0.61	
PostMI	45.00 (19.61)	55.80 (17.32)	0.07	0.58
PreRHB	31.95 (21.31)	44.75 (20.09)	0.58	0.61
PostRHB	22.20 (17.27)	35.98 (19.09)	0.02	0.75

Base: baseline; postMI: post-motor imagery; preRHB: pre-rehabilitation; postRHB: post-rehabilitation; CI: confidence interval; DD: tip-to-tip pinch; LT: lateral pinch; TD: three-point pinch; VAS: Visual Analogue Scale; DASH: Disabilities of the Arm, Shoulder, and Hand; FP: functional pain; RT: rest pain; MCF: metacarpophalangeal; EG: experimental group; CG: control group.

**Table 4:** OR of participants who achieved the minimum clinically important difference (MCID) in self-reported variables in post-MI and post-BHR measurements.

Variable		Exceed MCID Baseline−v			Exceed MCID Baseline−postRHB		
		YES	NO	OR (95% CI)	YES	NO	OR (95% CI)
**VAS FP**	EG	13 (65%)	7 (35%)	16.7 * (2.97–93.88)	16 (80%)	4 (20%)	4 * (0.98–16.27)
	CG	2 (10%)	18 (90%)		10 (50%)	10 (50%)	
**VAS RP**	EG	8 (40%)	12 (60%)	1.55 (0.42–5.76)	13 (65%)	7 (35%)	2.27 (0.63–8.1)
	CG	6 (30%)	14 (70%)		9 (45%)	11 (55%)	
**DASH**	EG	4 (20%)	16 (80%)	2.2 (0.36–13.97)	16 (80%)	4 (20%)	7.4 * (1.77–31.04)
	CG	2 (10%)	18 (90%)		7 (35%)	13 (65%)	
**COCHIN**	EG	7 (35%)	13 (65%)	2.1 (0.51–9)	18 (90%)	2 (10%)	9 * (1.63–49.44)
	CG	4 (20%)	16 (80%)		10 (50%)	10 (50%)	

VAS: Visual Analogue Scale; FP: functional pain; RP: rest pain; DASH: Disabilities of the Arm, Shoulder, and Hand; EG: experimental group; CG: control group; OR: odds ratio; postMI: post-motor imagery; postRHB: post-rehabilitation. * *p* < 0.05.

## Data Availability

The datasets used and the data analyzed in this study will be made available upon reasonable request to the corresponding author (J.Á.D.-G.). The data are not publicly available due to ethical reasons.
